# Resistance to Antifungals in Non-albicans Candida Species Isolates in the Southeast Region

**DOI:** 10.1017/ash.2024.228

**Published:** 2024-09-16

**Authors:** Priscilla Pineda, Erin Hitchingham, Melphine Harriott, Cody Rocha, Cherie Drenzek, Grace Lee, Ashley Marin, Melanie Roderick, JoAnna Wagner, Carolyn Stover

**Affiliations:** Tennessee Department of Health; TN Department of Health HAI/AR Program; Georgia Department of Public Health; LOUISIANA DEPARTMENT OF HEALTH - OFFICE OF PUBLIC HEALTH; Louisiana Department of Health; Alabama Department of Public Health

## Abstract

**Background:** Antimicrobial resistance is a growing problem in Candida spp., leading to treatment challenges and increased morbidity and mortality. The World Health Organization (WHO) fungal priority pathogens list classifies C. glabrata, C. tropicalis, and C. parapsilosis as high priority and leading causes of candidemia with high fluconazole resistance. In the US, these organisms are the most frequently isolated non-albicans Candida species. In 2016, the Antibiotic Resistance Laboratory Network (ARLN) was created to monitor resistance threats, including in Candida spp. This study describes the proportion of resistance in C. glabrata, C. parapsilosis, and C. tropicalis isolates sent to the Southeast ARLN from 2017 to 2023. **Methods:** This study evaluated C. glabrata, C. parapsilosis, and C. tropicalis submitted to the Southeast ARLN from Alabama, Florida, Georgia, Louisiana, Mississippi, and Tennessee from February 2017- September 2023. Species identification was confirmed by Bruker Biotyper matrix assisted laser desorption-ionization time of flight (MALDI-TOF). Antifungal susceptibility testing (AFST) was performed using TREK frozen broth microdilution panels. Minimum inhibitory concentration values from the clinical instrument were used to determine susceptibility based on Clinical and Laboratory Standards Institute (CLSI) standard interpretations from the 2020 CLSI M60 guidelines. Data were extracted from the laboratory information management system. Analyses were conducted using SAS v9.4. **Results:** AFST testing was performed on 660 C. glabrata, 500 C. parapsilosis, and 233 C. tropicalis isolates from within the Southeast region. The predominant specimen sources by species were blood 25.30% C. glabrata; other/not specified 27.80% C. parapsilosis; and lower respiratory 36.91% C. tropicalis. Resistance to fluconazole is as follows: C. glabrata, 12.88%; C. parapsilosis, 3.41%; C. tropicalis, 36.64%. Resistance to voriconazole is as follows: C. parapsilosis, 1.00%; C. tropicalis 30.04%. Resistance to at least one echinocandin (Anidulafungin, Capsofungin, Micafungin) is as follows: C. glabrata, 1.67%; C. parapsilosis, 0.60%; C. tropicalis, 0.43%. Overall, there was a decreasing trend in resistance to fluconazole, and voriconazole in all three species between 2017 and 2023. **Conclusions:** Antifungal resistance in non-albicans Candida species represents an emerging public health threat, however, within the Southeast region, ARLN data has shown a decreasing trend of azole resistance. This may be due in part to changes in reporting requirements and submission criteria from within the region. Nevertheless, C. tropicalis showed high resistance to azoles within the Southeast region. These Candida species should be monitored to inform clinical decision making and identify resistance patterns in other US regions due to their increase in resistance worldwide.

